# Socio-demographic and clinical characteristics of type 1 diabetes patients associated with emergency room visits and hospitalizations in Mexico

**DOI:** 10.1186/s12913-018-3412-3

**Published:** 2018-08-03

**Authors:** Svetlana V. Doubova, Aldo Ferreira-Hermosillo, Ricardo Pérez-Cuevas, Casper Barsoe, Erick Gryzbowski-Gainza, Juan E. Valencia

**Affiliations:** 1 Epidemiology and Health Services Research Unit, CMN Siglo XXI, Mexican Institute of Social Security, Av. Cuauhtemoc 330, Col. Doctores, 06720 Mexico City, Mexico; 2 Unidad de Investigación en Endocrinología Experimental, Hospital de Especialidades del CMN siglo XXI, Mexico City, Mexico; 3 Center for Health Systems Research, National Institute of Public Health, Universidad No. 655 Colonia Santa María Ahuacatitlán, 62100 Cuernavaca, Mexico; 4 Diabetes Health Economics & Reimbursement, Medtronic, Devonshire St 18000, Northridge, CA 91325-1219 USA; 5 Medtronic, Insurgentes Sur 863, Colonia Napoles, 03810 Ciudad de Mexico, Mexico; 6 Diabetes Health Economics & Reimbursement, Medtronic, NW 41st Street 9850, Miami, FL 33178 USA

## Abstract

**Background:**

To describe the demographic and clinical characteristics of Type 1 diabetes (T1D) patients affiliated with the Mexican Institute of Social Security (IMSS) and ascertain the socio-demographic and clinical risk factors associated with emergency room (ER) visits and diabetes-related hospitalizations.

**Methods:**

We conducted secondary data analysis of a cross-sectional study. The study included T1D patients 18 years of age and older who in 2016 attended follow-up visits at the endocrinology department of two IMSS tertiary care hospitals in Mexico City. The study variables included demographics, acute and chronic complications, and healthcare services utilization. Multiple Poisson and negative binomial regressions served to determine the association between the study covariates and the dependent variables: ER visits and diabetes-related hospitalizations.

**Results:**

The study included 192 patients, of which 29.2% were men; average age was 32.3 years, with only 13.6% controlled (glycosylated hemoglobin (HbA1C) < 7%); the mean HbA1C was 9.2, and 64.6% presented chronic complications. During 2016, 39.0% visited ER services, and 33.9% were hospitalized. The common risk factors for ER visits and hospitalization were older age at the beginning of diabetes, severe acute complications, chronic microvascular and macrovascular complications, and other comorbidities. Female sex, high school education, depression, and repeated visits to the endocrinologist were associated with ER visits, whereas active smoking and the interaction between diabetes duration > 10 years and HbA1c > 9.0% were additional risk factors for hospitalization.

**Conclusion:**

The poor clinical conditions of T1D patients contribute to explain the escalating demand for health services for diabetes patients at the IMSS. The identification of risk factors enables focalizing interventions to improve the health outcomes of T1D patients and reduce the proportion of ER visits and hospital admissions.

## Background

Type 1 diabetes (T1D) is a heterogeneous disorder characterized by the destruction of pancreatic beta cells, resulting in absolute insulin deficiency [1]. It usually develops in childhood; yet, it can occur at any age [2], with up to one-fourth of T1D diagnosed during adulthood [3]. Worldwide, T1D comprises 5–10% of the total number of cases of diabetes [4].

T1D is a life-long chronic disease associated with the development of acute (i.e. ketoacidosis, hypoglycemic episodes) and micro- and macrovascular complications. Microvascular complications include nephropathy, retinopathy and neuropathy. Macrovascular complications are coronary artery disease, peripheral arterial disease, and stroke. T1D patients have a ten-fold risk for cardiovascular events compared to age-matched non-diabetic populations [5]. Also, progressive kidney disease and retinopathy are more frequent in T1D than in Type 2 Diabetes (T2D) patients [6, 7]. The incidence of these complications depends on the duration of the disease. The highest incidence rate (3% per year) of progressive kidney disease has been reported 10 to 20 years after the onset of diabetes. Approximately one-third of T1D patients develop microalbuminuria after 15 years of disease progression; of whom, less than half develop nephropathy. The incidence of diabetic retinopathy rises to 14.7% in patients with < 5 years duration and can reach 81% after 20 years [6, 7]. Diabetic polyneuropathy is another frequent complication; it reaches up to 50% prevalence, causing neuropathic pain and disability due to foot ulceration and amputation [8]. The life expectancy of T1D patients is 11–13 years less compared to the general population [9, 10]. T1D patients face a two-fold risk of death from any cause in comparison to matched controls, and the risk increases with elevated levels of glycated hemoglobin (HbA1c) and is highest for patients with HbA1c > 9% [11].

The extensive variety and severity of clinical manifestations of T1D increase the probability of emergency room (ER) visits and hospital admissions. The most frequently reported causes of ER visits are severe hypoglycemia [12], diabetic ketoacidosis, and neurological complications [13]. A recent systematic review of 32 studies conducted in developed countries stressed that T1D patients are three times more likely to be hospitalized than non-diabetic patients and stay in the hospital twice as long [14]. The clinical and sociodemographic risk factors associated with increased odds of hospital admission in T1D patients are the following: being female [15, 16], low socioeconomic status [17] HbA1c > 9% [18–23], chronic complications of diabetes [24], and comorbidities (e.g., hypertension) [21].

In developing countries, the scarcity of studies on utilization patterns of emergency care and hospital admission of T1D patients justifies examining the situation. Mexico is an appropriate case for review since the prevalence of diabetes is escalating and taking a heavy toll. The Mexican Institute of Social Security (IMSS), the largest social security provider in the country, reports periodically on the magnitude of this challenge. IMSS is a contributory (through payroll taxes) public institution. In 2012, IMSS informed that between 2000 and 2010, the number of new T1D cases rose from 3.4 to 6.2 per 10,000 insured persons under 19 years of age [25]. In 2016, IMSS had 62 million affiliates that included 3.8 million patients with diabetes (T1D and T2D) [26].

IMSS is facing financial and health delivery-related challenges to keep up with the needs of diabetes patients. This institution provides a comprehensive bundled set of healthcare, economic and social benefits to workers (and their families) of the formal labor market.

The first challenge is the financial impact of diabetes. IMSS provides unlimited healthcare benefits that include primary and secondary prevention, hospital and rehabilitation care, orthopedic devices and medicines. Therefore, the upward trend in prevalence and utilization of medical care of chronic patients is causing health costs to climb. In 2017, IMSS reported that diabetes was among the top six conditions (cardiovascular disease and hypertension, diabetes, cervical, breast and prostate cancer, and chronic renal insufficiency) causing high health outlays [27].

The second challenge is the economic burden from indirect costs, since IMSS affiliates are entitled to receive temporary and permanent disability leave and pensions. Between 2000 and 2013, diabetes-related pensions almost doubled, from USD$58.28 million to USD$111.62 million [28]. From an economic and social perspective, the poor health of T1D patients −particularly chronic complications− have critical consequences such as increased work absence, reduced work productivity, permanent occupational disability, early retirement, shortened life-expectancy and low quality-of-life [29, 30].

The third challenge that IMSS faces is the high demand for healthcare. At IMSS diabetes is the second cause of visits to primary care clinics, fifth cause of visits to specialized ambulatory care, and among the top causes of hospital discharge, death, and disability. Half of the patients undergoing dialysis have diabetes [31]. In 2013, the rate of minor amputations was 162.5 per 100,000, and of major amputations was 111.1 per 100,000 diabetes patients. The average age at the time of the amputation was 65.6 years [32]. The analysis of a retrospective cohort of 34,014 Mexican workers with permanent occupational disability caused by T1D and T2D during the years 2000–2013 at IMSS found that the mean age for permanent occupational disability was 51.6 years. Life expectancy was 7.26 years less, and 55.5 ± 8.0 years (mean, SD) of age at death [28]. Renal complications are the principal characteristic associated with a significantly elevated all-cause mortality (Hazard Ratio 3.49; 95% CI 3.18–3.83) [28]. Assessing the risks for ER visits and hospital admission of T1D patients is justifiable since there is a lack of evidence in developing countries such as Mexico that can guide interventions to achieve glycemic control, reduce acute complications and delay chronic complications.

The objectives of the study were to describe the demographic and clinical characteristics of T1D patients affiliated with IMSS and ascertain the socio-demographic and clinical risk factors associated with emergency room (ER) visits and diabetes-related hospitalizations.

## Methods

A secondary data analysis of the baseline characteristics of the group of T1D patients included in the health-economic evaluation study of diabetic treatment at IMSS was performed. The study included 192 T1D patients 18 years of age and older who in 2016 attended follow-up visits at the endocrinology department of two IMSS tertiary care hospitals in Mexico City. These hospitals were selected by convenience, since they are the largest in Mexico City, and because, according to IMSS regulations, is in this type of settings where endocrinologists must provide healthcare to T1D patients. From February to May 2017, two trained research nurses collected the information from clinical records and telephone-interviews of T1D patients who fulfilled the inclusion criteria. The interviews verified and complemented clinical records lacking information. The IMSS Research and Ethics Committee approved the study (No. R 2016–785-091).

The study included the following variables:1) General characteristics: sex, age, schooling, occupation, smoking (never smoke, active smoking, smoked but quit smoking), and regular alcohol consumption (considered up to 7 standard drinks per week for women and 14 standard drinks per week for men. A standard drink was defined as any drink containing 14 g of pure alcohol, as recommended by the National Institute of Alcohol Abuse and Alcoholism of the United States. In the study population, no one drank more than seven drinks a week that was considered as light to moderate alcohol consumption.

2) Clinical characteristics: age at diagnosis of T1D, duration of the disease, body mass index (BMI: kg/m^2^), overweight or obesity (BMI ≥ 25 kg/m^2^), systolic and diastolic blood pressure, HbA1c, total high-density lipoprotein (HDL) and low-density lipoprotein (LDL) cholesterol, triglycerides, creatinine clearance, protein in a 24-h urine sample and serum creatinine. T1D severe acute complications comprised diabetic ketoacidosis and severe hypoglycemia that required ER visit or hospitalization. Chronic microvascular complications (nephropathy, retinopathy, neuropathy) and chronic macrovascular complications (coronary artery disease, peripheral arterial disease, and stroke); comorbidity (hypertension, depression and others); type of insulin treatment (insulin monotherapy, combination of intermediate and fast-acting insulin and combination of fast-acting and long-acting insulin), number of visits to the endocrinologist, ER visits and hospitalizations in 2016.

Dependent variables: number of emergency room visits and diabetes-related hospitalizations in 2016.

### Sample size and statistical analysis

The sample size was based on the practice of ensuring at least 10 participants per each covariate included in the multiple regression analysis [33].

We used descriptive statistics to analyze the patients’ characteristics. To ascertain the socio-demographic and clinical risk factors associated with the higher ER visits and diabetes-related hospitalizations; first, we evaluated the presence of overdispersion in the outcome variables. We found that the mean of the use of the ER visits was: 0.6145833, the variance was 0.87685428 and the dispersion was 1.43. The mean of hospital admissions due to diabetes was 0.5520833, the variance was 1.6517234 and the dispersion was 2.99. Then, to consider the overdispersion and clustering of the observations within two clusters (hospitals) we used the original count variables and built four types of models (1) Poisson regression model with cluster-robust standard errors; 2) Negative binomial regression model with cluster-robust standard errors; and 3) Zero-inflated Poisson and (4) Zero-inflated negative binomial regression models, considering lots of zeros in the data and potential existence of different unobservable reasons in the use of emergency room and hospitalizations between men and women. The comparison of the models were based on the Akaike’s and Bayesian information criteria and on the goodness-of-fit chi-squared test for Poisson regression model and vuong test to compare the zero-inflated model to a standard models (Poisson, or negative binomial regression). The comparison of the models revealed that in the case of the ER visits the above-mentioned criteria were better for the Poisson regression model with cluster-robust standard errors and in the case of the hospitalizations these criteria were better for the negative binomial regression model with cluster-robust standard errors. To build the models we included all conceptually and clinically relevant variables that we identified through the literature review. Also, we tested possible interactions between diabetes duration##HbA1C levels; as well as, T1D complications##HbA1C levels. We found, statistically significant interactions between severe acute diabetic complications and HbA1C in the case of the ER visits model and duration of diabetes (> 10 years) and HbA1C levels (> 9%) in the case of the hospitalization model.

Finally, from 192 patients who participated in the study, eight (4.2%) had missing data in one or more of the study variables; therefore, we excluded these eight patients from the multiple regression analysis, as a missing rate of 5% or less is usually considered not significant and does not require missing data treatment [34]. We used Stata 14.0 (Stata Corp, College Station, Texas, United States) for the statistical analysis; *p* < 0.05 was considered statistically significant.

## Results

From 207 patients with T1D who in 2016 visited the out-patient services of the endocrinology department of participating hospitals, 192 (92.8%) were included. We did not include 15 patients (7.2%) because of incomplete information in clinical records and/or lack of contact information.

Table 1 describes the demographic characteristics and the cigarette and alcohol consumption of the study population. Out of the 192 patients, 29.2% were men; the average age was 32.3 years; 44.3% had completed high school, and 42.7% had a university degree. Regarding employment status, 23.4% were unemployed, 21.9% were semi-skilled, and 20.8% were considered skilled labor; 7.8% were pensioned or retired. Only 9.9% reported active smoking, while 63.5% answered that they had never smoked or quit smoking (24%); 11.5% reported light to moderate alcohol consumption.

**Table 1:** Demographic characteristics, cigarettes and alcohol consumption among patients with type 1 diabetes affiliated to the Mexican Institute of Social Security

	*N* = 192 (%)
Demographic characteristics	
Male sex	29.2
Current age, years, mean (Standard Deviation)	32.3 (10.8)
Age-groups	
≤ 20 years	13.0
21–30 years	35.4
31–40 years	30.2
> 40 years	21.4
Schooling	
Secondary school or less	12.0
High school or technical or commercial career	44.3
University degree or higher	42.7
Missing data	1.0
Occupation	
Student	19.8
Unemployment/housekeeping	23.4
Unskilled labor	4.2
Semi-skilled labor	21.9
Skilled labor	20.8
Retired or pensioned	7.8
Missing data	2.1
Alcohol and tobacco consumption	*N* = 192
Smoking	
Never smoked	63.5
Smoked, but quit smoking	24.0
Active smoking	9.9
Missing data	2.6
Regular light to moderate alcohol consumption	
Yes	11.5
No	84.3
Missing data	4.2

Table 2 depicts the patients’ clinical characteristics. The mean age at diabetes diagnosis was 13.8 years; only 19.3% have had diabetes for less than 10 years and 49.5% had normal weight, while 3.1% were underweight, 34.9% were overweight and 12.5% were obese. The mean systolic and diastolic blood pressure figures were 107 and 70.9 mmHg, respectively, while the mean HbA1C was 9.2%; only 13.6% had HbA1C < 7%, while 46.6% had HbA1C > 9%. On average, triglycerides, total cholesterol, HDL and LDL cholesterol were within acceptable limits. 173 patients had data on creatinine clearance with a mean value for men of 79.4 mL/min and women of 74.4 mL/min; these figures were below the range considered acceptable (97–137 mL/min for healthy men and 88–128 mL/min for healthy women). According to the medical diagnosis, 35.4% did not have chronic complications of diabetes; among those with complications, 26% had one, 21.4% had two, and 17.2% had three or more. Chronic microvascular complications were more frequent (64.1%) than macrovascular complications (8.3%). During 2016, 19.3% presented acute diabetic complications, such as severe hypoglycemia (11.9%) or ketoacidosis (8.3%) Some patients presented either one or both of these complications. Furthermore, most patients had comorbidities such as hypertension (29.7%), depression (5.7%), or other comorbidities such as hypothyroidism and diseases of the musculoskeletal system (50.5%).

**Table 2:** Clinical characteristics, use of the emergency room and hospitalizations among patients with type 1 diabetes affiliated to the Mexican Institute of Social Security

	*N* = 192 (%)
Clinical characteristics	
Age at the beginning of diabetes, years, mean (Standard Deviation -SD-)	13.8 (6.6)
Diabetes duration	
< 10 years	19.3
≥ 10 years < 15 years	21.3
≥ 15 years < 20 years	18.2
≥ 20 years	14.2
BMI (kg/m2), mean (SD)	25.1 (4.3)
Nutritional status	
Underweight < 18.5 kg/m^2^	3.1
Normal weight (BMI 18.5–24.9 kg/m^2^)	49.5
Overweight (BMI 25.0–29.9 kg/m^2^)	34.9
Obesity (BMI of 30 or higher kg/m^2^)	12.5
Systolic Blood Pressure, mmHg (SD)	107.0 (15.3)
Diastolic blood pressure, mmHg (SD)	70.9 (9.6)
HbA1c, %, mean (SD)	9.2 (2.2)
HbA1c < 7%	13.6
HbA1c > 9%	46.6
Triglyceride, mg/dl, mean (SD)	145.7 (185.2)
Total Cholesterol, mg/dl, mean (SD)	179.7 (46.8)
HDL, mg/dl, mean (SD)	
Men	*N* = 54: 49.1 (16.5)
Women	*N* = 128: 53.2 (16.6)
LDL, mg/dl, mean (SD)	100.9 (35.9)
Creatinine clearance, mL/min, mean (SD)	
Men	*N* = 51: 79.4 (40.7)
Women	*N* = 122: 74.4 (37.1)
Diabetic complications	
Number of diabetic chronic complications	
Without diabetic chronic complications	35.4
One diabetic complication	26.0
Two diabetic complications	21.4
Three or more diabetic complications	17.2
Microvascular chronic complications^a^	64.1
Nephropathy	41.2
Retinopathy	40.6
Neuropathy	30.2
Foot ulcers and amputations	5.7
Macrovascular chronic complications	8.3
Severe acute diabetic complications during 2016^a^	19.3
Severe hypoglycemia	11.9
Ketoacidosis	8.3
Comorbidity	
Hypertension	29.7
Depression	5.7
Other	50.5
Insulin therapy	
Insulin monotherapy	16.1
Combination of intermediate and fast acting insulin	47.4
Combination of fast-acting and long-acting insulin	36.5
Number of consultations with endocrinologist, mean (SD)	3.14 (1.5)
Use of the emergency room and hospitalizations during 2016	
Visits to the emergency room	
0	61.0
1	23.4
2	10.9
≥ 3	4.7
Causes of the emergency room visits^a^	*N* = 75
Hyperglycemia	29.3
Hypoglycemia	26.7
Acute nephritic syndrome	6.7
Acute urinary infection	6.7
Other causes related to diabetes	2.7
Causes not related to diabetes	28.0
Number of hospital admissions	*N* = 192
0	66.1
1	21.9
2	5.7
≥ 3	6.3
Causes of hospitalizations^a^	*N* = 65
Hyperglycemia	24.6
Ketoacidosis	21.5
Hypoglycemia	13.8
Due to chronic diabetes complications	26.2
Causes not related to diabetes	13.8
Duration of hospitalizations, days, mean (SD)	10.7 (10.1)

^a^The chronic microvascular complications or severe acute diabetic complications are presented as percentages; however, the sum of the percentages of each type of complication does not add up to 100%, as patients can have more than one acute or chronic complications, since these complications are not mutually exclusive. The same rule is applicable for the causes of emergency room visits and diabetes-related hospitalizations

All patients were prescribed insulin: 16.1% received insulin monotherapy, 47.4% received a combination of intermediate-acting and fast-acting insulin and 36.5% a combination of fast-acting and long-acting insulin.

During 2016, T1D patients had on average three consultations with the endocrinologist; 39% visited the ER (23.4% one time, 10.9% two times and 4.7% three or more times). The most frequent causes of ER visits were hyperglycemia (29.3%) and hypoglycemia (26.7%). Also, 33.9% were hospitalized (21.9% one time, 5.7% two times and 6.3% three or more times). The main reasons were hyperglycemia (24.6%), ketoacidosis (21.5%), hypoglycemia (13.8%), chronic diabetes complications (26.2%), and other diagnoses unrelated to diabetes (13.8%). The average length of hospital stay was 10.7 days.

Table 3 shows the results of the Poisson regression model with cluster-robust standard errors for sociodemograhic and clinical characteristics associated with ER visits. The coefficients represent prevalence ratios (PR); their interpretation is the same as for the risk ratios. The multivariate analysis revealed that being female (adjusted PR:1.52; 95% CI:1.51–1.53), older age at the beginning of diabetes (adjusted PR:1.01; 95% CI:1.01–1.02), high school (adjusted PR:1.69; 95% CI:1.41–2.02) or university degree or higher (adjusted PR:2.18; 95% CI:1.38–3.44), presence of severe acute diabetic complications (adjusted PR:7.54; 95% CI:4.07–13.98), chronic microvascular (adjusted PR:2.01; 95% CI:1.99–2.02) and macrovascular (adjusted PR:1.18; 95% CI:1.13–1.24) complications, depression (adjusted PR:1.72; 95% CI:1.00–2.94), other comorbidities (adjusted PR:1.27; 95% CI:1.26–1.29) and frequent visits to the endocrinologist (adjusted PR:1.09; 95% CI:1.06–1.11) increased the probability of a higher number of ER visits. The regular light to moderate alcohol consumption (adjusted PR:0.68; 95% CI:0.61–0.75), higher body mass index (adjusted PR:0.95; 95% CI:0.92–0.99) and treatment combination of intermediate and fast acting insulin (adjusted PR:0.89; 95% CI:0.81–0.97) were associated with a low probability of a higher number of ER visits. Also, we found the significant interaction between severe acute diabetic complications and higher levels of HbA1c (adjusted PR:0.93; 95% CI:0.90–0.95). This interraction shows that the effect of acute complication on ER visits depends on the value of HbA1C; therefore, for patients with higher HbA1C the effect of acute severe complications on ER visits is lower.

**Table 3:** Characteristics associated with the use of the emergency room in patients with type 1 diabetes (Poisson regression with cluster robust standard errors *n* = 184)

	Un-adjusted PR	[95% CI], p	Adjusted PR	[95% CI], p
General characteristics				
Male	Ref.		Ref.	
Female	**1.39**	**[1.36, 1.41], 0.000**	**1.52**	**[1.51, 1.53], 0.000**
Age at the beginning of diabetes (years)	1.01	[0.99, 1.03], 0.302	**1.01**	**[1.01, 1.02], 0.000**
Schooling				
Secondary school or less	Ref.		Ref.	
High school or technical or commercial career	0.95	[0.65, 1.39], 0.781	**1.69**	**[1.41, 2.02], 0.000**
University degree or higher	0.81	[0.65, 1.00], 0.050	**2.18**	**[1.38, 3.44], 0.001**
Active smoking				
No	Ref		Ref.	
Yes	1.20	[0.70, 2.06], 0.504	1.28	[0.72, 2.30], 0.402
Regular light to moderate alcohol consumption				
No	Ref		Ref	
Yes	**0.56**	**[0.44, 0.72], 0.000**	**0.68**	**[0.61, 0.75], 0.000**
Clinical characteristics				
Diabetes duration, years	0.99	[0.96, 1.04], 0.869	0.98	[0.96, 1.01], 0.118
HbA1C (%)	0.98	[0.89, 1.08], 0.717	0.95	[0.86, 1.04], 0.258
Severe acute diabetic complications				
No	Ref.		Ref.	
Yes	**4.05**	**[2.00, 8.20], 0.000**	**7.54**	**[4.07, 13.98], 0.000**
Severe acute diabetic complications## HbA1C	0.99	[0.87, 1.13], 0.927	**0.93**	**[0.90, 0.95], 0.000**
Microvascular chronic complications				
No	Ref.		Ref.	
Yes	**1.89**	**[1.19, 3.01], 0.007**	**2.01**	**[1.99, 2.02], 0.000**
Macrovascular chronic complications				
No	Ref.		Ref.	
Yes	1.60	[0.81, 3.16], 0.174	**1.18**	**[1.13, 1.24], 0.000**
Depression				
No	Ref.		Ref.	
Yes	2.21	[0.84, 5.83], 0.107	**1.72**	**[1.00, 2.94], 0.049**
Other comorbidities				
No	Ref.		Ref.	
Yes	**1.43**	**[1.33, 1.54], 0.000**	**1.27**	**[1.26, 1.29], 0.000**
Body mass index (kg/m2)	**0.94**	**[0.92, 0.96], 0.000**	**0.95**	**[0.92, 0.99], 0.013**
Insulin therapy				
Insulin monotherapy	Ref.		Ref.	
Combination of intermediate and fast acting insulin	**0.76**	**[0.74, 0.77], 0.000**	**0.89**	**[0.81, 0.97], 0.008**
Combination of fast-acting and long-acting insulin	0.85	[0.50, 1.43], 0.536	1.05	[0.63, 1.75], 0.857
Number of consultations with endocrinologist	**1.13**	**[1.06, 1.20], 0.000**	**1.09**	**[1.06, 1.11], 0.000**

*PR* prevalence ratios, *CI* confidence interval. The bold values highlight the statistically significant PR

Table 4 depicts the results of the Negative binomial regression with cluster-robust standard errors model for patient characteristics associated with diabetes-related hospital admissions. The analysis indicates that older age at the beginning of diabetes (adjusted PR:1.05; 95% CI:1.04–1.05), active smoking (adjusted PR:2.74; 95% CI:2.58–2.91), severe acute complications (adjusted PR:3.57; 95% CI:2.05–6.22), chronic microvascular (adjusted PR:7.04; 95% CI:3.72–13.32) and macrovascular complications (adjusted PR:2.32; 95% CI:1.02–5.25) and other comorbidities (adjusted PR:1.91; 95% CI:1.09–3.35) increased the probability of a higher number of hospitalizations. Also, we found the significant interaction between diabetes duration > 10 years and HbA1c > 9.0% (adjusted PR:1.64; 95% CI:1.26–2.13). This interaction shows that the effect of diabetes duration on hospitalizations depends on the value of HbA1C; particularly, for patients with the HbA1c > 9.0% the effect of prolonged diabetes duration (> 10 years) on hospitalizations is higher.

**Table 4:** Characteristics associated with diabetes-related hospitalizations in patients with type 1 diabetes (Negative binomial regression with cluster robust standard errors *n* = 184)

	Un-adjusted PR	[95% CI], p	Adjusted PR	[95% CI], p
General characteristics				
Male	Ref.		Ref.	
Female	0.71	[0.32, 1.46], 0.349	0.77	[0.55, 1.45], 0.456
Age at the beginning of diabetes (years)	1.04	[0.97, 1.11], 0.245	**1.05**	**[1.04, 1.05], 0.000**
Schooling				
Secondary school or less	Ref.		Ref.	
High school or technical or commercial career	**0.73**	**[0.72, 0.74], 0.000**	1.64	[0.69, 3.87], 0.264
University degree or higher	0.53	[0.22, 1.30], 0.168	1.78	[0.45, 7.13], 0.412
Active smoking				
No	Ref.		Ref.	
Yes	1.24	[0.75, 2.03], 0.402	**2.74**	**[2.58, 2.91], 0.000**
Regular light to moderate alcohol consumption				
No	Ref.		Ref.	
Yes	**0.30**	**[0.23, 0.42], 0.000**	**0.31**	**[0.305, 0.32], 0.000**
Clinical characteristics				
Diabetes duration > 10 years	**0.30**	**[0.06, 1.46], 0.136**	0.23	[0.07, 1.27], 0.100
HbA1c ≤ 7%	Ref		Ref	
HbA1c 7.1–9%	**0.47**	**[0.42, 0.52], 0.000**	1.22	[0.84, 1.78], 0.289
HbA1c > 9.0%	0.78	[0.51, 1.19], 0.250	**0.46**	**[0.22, 0.95], 0.036**
Diabetes duration > 10 years##	**3.48**	**[1.01, 12.07], 0.049**	1.19	[0.43, 3.31], 0.742
HbA1c 7.1–9%				
Diabetes duration > 10 years##	1.14	[0.64, 2.02], 0.651	**1.64**	[**1.26, 2.13], 0.000**
HbA1c > 9.0%				
Severe acute diabetic complications				
No	Ref.		Ref.	
Yes	**3.34**	**[2.22, 5.02], 0.000**	**3.57**	**[2.05, 6.22], 0.000**
Microvascular chronic complications				
No	Ref.		Ref.	
Yes	**4.01**	**[3.59, 4.49], 0.000**	**7.04**	**[3.72, 13.32], 0.000**
Macrovascular chronic complications				
No	Ref.		Ref.	
Yes	1.81	[0.44, 7.38], 0.406	**2.32**	**[1.02, 5.25], 0.044**
Depression				
No	Ref.		Ref.	
Yes	2.50	[0.81, 7.72], 0.110	0.85	[0.03, 20.79], 0.922
Other comorbidities				
No	Ref.		Ref.	
Yes	**1.68**	**[1.54, 1.84], 0.000**	**1.91**	**[1.09, 3.35], 0.024**
Nutritional status				
Underweight, or normal weight (BMI < 24.9)	Ref		Ref	
Overweight (BMI 25.0–29.9 kg/m^2^)	0.47	[0.16, 1.37], 0.167	**0.32**	**[0.19, 0.52], 0.000**
Obesity (BMI of 30 or higher kg/m^2^)	0.27	[0.02, 3.47], 0.317	0.14	[0.02, 1.04], 0.055
Insulin therapy				
Insulin monotherapy	Ref.		Ref.	
Combination of intermediate and fast acting insulins	0.49	[0.21, 1.16], 0.106	**0.64**	**[0.51, 0.81], 0.000**
Combination of fast-acting and long-acting insulins	0.53	[0.24, 1.19], 0.125	**0.52**	**[0.37, 0.72], 0.000**
Number of consultations with endocrinologist	1.09	[0.97, 1.23], 0.150	0.99	[0.79, 1.25], 0.978

*PR* prevalence ratios, *CI* confidence interval. The bold values highlight the statistically significant PR

At the same time, regular light to moderate alcohol consumption (adjusted PR:0.31; 95% CI:0.31–0.32), overweight (PR:0.32; 95% CI:0.19–0.52) and treatment with combination of intermediate and fast acting insulin (adjusted PR:0.64; 95% CI:0.51–0.81) and with combination of fast and long-acting insulin (adjusted PR:0.52; 95% CI:0.37–0.72) decreased the probability of being hospitalized more frequently.

## Discussion

The main findings of the present study indicate that a high proportion of T1D patients had poor glycemic control and acute and chronic complications; these patients visited the ER (39%) and were hospitalized (33.9%) very often in 1 year. The results contribute to explain the escalating demand for health services of diabetes patients at IMSS. Older age at the diagnosis of diabetes, severe acute complications, chronic microvascular and macrovascular complications, and other comorbidities increase the risk of visiting the ER and being hospitalized. In particular, female sex, high school education, depression, and repeated visits to the endocrinologist were associated with ER visits; whereas active smoking and the interaction between diabetes duration > 10 years and HbA1c > 9.0% were additional risk factors for hospitalization.

Poor glycemic control and the high proportion of diabetes complications signal poor performance of health services to care for T1D patients. The goal of the treatment of T1D is to achieve normo-glycaemia to avoid complications [35]. In our study, only 13.6% of patients had HbA1C < 7%, and the mean HbA1C was 9.2%. These figures are far higher from those reported in other countries. The EURODIAB Prospective Complications Study from 16 European countries that included 3250 T1D patients reported that the mean HbA1c was 8.4% [36]. Another study in Finland that included 2107 T1D patients found that the mean HbA1c was 8.5% [37]. A study in Colombia of 217 T1D patients reported that the mean HbA1c was 8.9, and 45.6% of patients had HbA1C < 7% [38].

There is a relationship between high levels of HbA1C and hospitalization [18–23]. The results of the present study show a significant interaction between diabetes duration > 10 years and HbA1c > 9.0%. This finding signals that in patients with HbA1c > 9.0%, the effect of prolonged diabetes duration (> 10 years) on the frequency of hospitalizations is higher.

There were more patients with chronic complications in our sample than in other countries. Cohort studies of T1D patients in the Diabetes Control and Complications Trial (DDCT) indicate that after 30 years of disease progression the cumulative incidence of chronic complications was as follows: retinopathy (47%), nephropathy (17%), and cardiovascular disease (14%) [39]. The study from Colombia mentioned before, reported lower percentages: retinopathy 28.2%, nephropathy 22.1%, and neuropathy 23.5%. By comparison, in our study, retinopathy was 40.6%, nephropathy 41.2%, and neuropathy 30.2%.

There was a high proportion of emergency visits (39%) and diabetes-related hospital admissions (33.9%) due to acute and chronic complications. Different studies in Denmark [40], Peru [41], and Scotland [24] have reported that the proportion of T1D patients going to the emergency room and hospital admissions varies between 20 and 28%. The findings of our study allow inferring that the high demand for ER visits and hospitalizations for T1D patients indicate deficient quality of care; furthermore, the large percentage of patients with chronic complications signal that they have been receiving substandard healthcare for extended periods. T1D patients require an individualized healthcare plan, continuous monitoring, and assessment for acute and chronic complications. In our study, despite that endocrinologists at secondary and tertiary care hospitals provide care for T1D patients, it is unclear whether integrated and coordinated care from different disciplines for these patients is available. T1D patients must have access to medical providers with T1D expertise able to deliver proper management, which in turn contributes to prevent and delay acute and chronic complications. The findings should prompt additional studies and interventions to assess and improve the model of care for these patients.

Depression and other comorbidities are also associated with more frequent ER visits and hospitalizations. Similarly to our findings, the association between depression and frequent ER visits has been described previously for patients who attend primary care [42] and for several sub-populations (e.g., older adults [43], patients with non-specific abdominal pain [44]). This finding indicates the importance of providing comprehensive care to T1D patients that includes psychological support and treatment of mood disorders and other comorbidity.

Currently, there is a lack of evidence regarding the association between body mass index and emergency room use and hospitalizations in T1D patients. A study of T1D patients from the Swedish National Diabetes Registry (1998–2003) reported that severe obesity was strongly associated with hospitalizations due to heart failure in patients with type 1 diabetes [45]. In our study we found that overweight but not obesity was associated with lower possibility of diabetes-related hospitalizations in comparison with underweight and normal weight patients. It is reasonable to consider the need to conduct additional studies to determine the magnitude of the association between BMI and use of ER and hospital admissions in T1D patients.

Previous studies have reported that high quality of healthcare and the use of new technologies allow for glycemic control and prevent diabetic complications. One of such technologies is the continuous subcutaneous insulin infusion with insulin-pump that can achieve an important reduction of HbA1C when compared to the multiple daily insulin injections [46, 47]. Also, the use of a glucose sensor augmented pump (SAP) was proposed. The SAP allows for continuous monitoring of glucose and the interruption of the insulin infusion when the glucose level is below a previously defined threshold; therefore, there is better control of glucose levels, and consequently the number, severity and duration of hypoglycemic events decreases [48]. Currently, the insulin-pump is within IMSS approved therapeutic supplies, yet in our sample we did not find patients who had been given this prescription. Although, in the absence of SAP, we found that the use of insulin combinations (e.g., intermediate and fast acting insulin) showed the possibility to decrease the probability of using the emergency room and being hospitalized when compared to patients with insulin monotherapy.

Several socio-demographic factors are also related to the more frequent use of ER and diabetes-related hospitalizations. In congruence with previous studies, we found that women had a higher probability of using the ER [15, 16] than men; while active smoking was a risk factor for hospitalization [49, 50]. Also, in this study, patients with a higher level of education and those that visited the endocrinologist more often had a higher probability of visiting the ER. Previous studies have reported that patients with high school level or above are more aware of their health needs and can better recognize the alarm signs of acute complications, thus seek care at the ER [51]. It is reasonable to assume that the poor health conditions of most of the T1D patients in our sample prompt them to seek oftener being seen by the endocrinologist and going to the ER. This finding reinforces the notion that the quality of care must be improved for these patients. Adittionally, we found that regular light to moderate alcohol consumption was a factor that decreased the risk of ER visits and hospitalizations. To the best of our knowledge, this protective effect of alcohol has been described in older women [52]; this effect is opposite of the alchol abuse that causes frequent use of the health services [53].

The study has several limitations. This was a secondary data analysis of a cross-sectional study that does not allow for causal inferences. Due to the nature of the data and because of the database only had the information on socio-demographic and clinical individual determinants of the use, we could not assess some relevant health services characteristics (e.g., quality of the process of healthcare and the users perception of quality).

## Conclusions

Clinical conditions of T1D patients are not reaching the expected results; this poor outcome contributes to explain the increase in the use for health services of these patients at IMSS. The findings of the study allow assuming that the model of care should be revisited and the quality of healthcare needs to improve to provide effective care to T1D patients. The identification of the risk factors for emergency room visits and hospital admissions enables focalizing interventions to improve the health outcomes of T1D patients and reduce the proportion of those that would need to attend to ER visits or be hospitalized.

## Abbreviations

BMI: Body mass index
CI: Confidence interval
ER: Emergency room
HbA1c: Glycated hemoglobin
HDL: High-density lipoprotein
IMSS: Mexican Institute of Social Security
LDL: Low-density lipoprotein
PR: Prevalence ratios
SD: Standard Deviation
T1D: Type 1 diabetes
T2D: Type 2 diabetes
